# Criterion Validity of Radon Test Values Reported by a Commercial Laboratory versus the Environmental Protection Agency

**DOI:** 10.3390/ijerph19063615

**Published:** 2022-03-18

**Authors:** Gary G. Schwartz, Marilyn G. Klug, Mark R. Williamson, Heather M. Schwartz

**Affiliations:** 1 Department of Population Health, School of Medicine & Health Sciences, University of North Dakota, Grand Forks, ND 58202-9037, USA; marilyn.klug@und.edu (M.G.K.); mark.williamson@und.edu (M.R.W.); 2 Department of Communication Sciences and Disorders, College of Arts & Sciences, University of North Dakota, Grand Forks, ND 58202, USA; heather.m.schwartz@und.edu

**Keywords:** radon, epidemiology, criterion validity, county level

## Abstract

Objective: Radon exposure is a proven cause of lung cancer and is a possible cause of other diseases. Recently, several ecologic studies explored the correlation of county-wide incidence rates for non-lung cancers with residential radon levels, using radon data reported by a commercial laboratory. However, the validity of the commercial radon data, i.e., whether they are an accurate representation of the radon levels in the counties from which they were drawn, is unknown. Methods: We compared county-wide radon data from the commercial laboratory with corresponding measurements from the same counties reported previously by the Environmental Protection Agency (EPA). Matching data were available for four states, Iowa, North Dakota, Texas, and Wisconsin, and were compared by paired t-tests. Criterion validity of the commercial tests, i.e., how well the commercial data predicted the EPA data, was tested using non-parametric methods, Kendall’s tau, Lin’s concordance, and Passing–Bablok regression. Results: The commercial and EPA data pairs from the four states were significantly positively correlated, although the size of the correlations was modest (tau = 0.490, Lin = 0.600). Passing–Bablok regression indicated that the commercial radon values were significantly higher than their EPA pairs and significantly overestimated radon at low levels (<4 pCi/L, *p* < 0.001). Conclusions: The commercial laboratory data were moderately predictive of EPA radon levels at the county level but were significantly biased upwards at low levels. The disagreement likely has several causes, including selection bias from homes that were tested voluntarily. Ecologic studies that employ radon data obtained from commercial laboratories should be interpreted with caution.

## 1. Introduction

Radon is a naturally occurring radioactive gas that results from the natural decay of uranium, thorium or radium present in rocks and soil. Radon can enter homes and accumulate there, especially during cold weather when homes are sealed. Radon is an established cause of lung cancer, accounting for approximately 22,000 lung cancer deaths per year in the U.S. [1]. Accordingly, the Environmental Protection Agency (EPA) advises individuals to test their homes for radon and to consider remediating their homes if the radon levels exceed 4 pCi/L (1 pCi/L = 37 Bq/m^3^).

The only disease for which there is a proven, etiologic role for radon is lung cancer, the evidence for which comes from occupational studies of miners and numerous case-control studies [2]. However, radon has been suggested as a possible cause of many other diseases, ranging from extra-pulmonary cancers to Alzheimer’s disease [3,4,5]. Authors seeking preliminary evidence for a role for radon in these diseases typically perform an ecologic study in which existing data on disease rates in specific geographic locations, e.g., states or counties, are correlated with corresponding radon data for those locations. There are two sources of extant radon data for such studies: the EPA and commercial laboratories. In 1993, the EPA published state and county maps of radon zones to identify areas with high potential for elevated indoor radon. The U.S. maps reflect data from yearlong radon tests in homes selected using scientific sampling methods [6,7]. The EPA also supported state-level studies that employed similar sampling methods and short-term radon tests, the type most commonly employed by home test kits [8]. In contrast, several companies that sell home radon test kits (e.g., Air Chek) report average (aggregate) radon values for counties where their tests have been used.

Commercial radon data at the county level have been used in several ecologic studies [9,10]. However, the validity of those data, i.e., whether they are a true representation of the radon values in the counties from which they were derived, is unknown. There are reasons to suspect that the data from commercial labs and the EPA would differ. Firstly, the commercial data are not derived from a random sample of homes. Secondly, the commercial data include duplicates tests from the same homes. For example, before undertaking home remediation, the EPA advises individuals with a high radon test result to confirm it with a second test. Additionally, individuals who remediate their homes are advised to re-test post-remediation [11]. In the first instance, the likely result would be a multiplication of high radon values; in the second, both high and low values would occur. Because the commercially available data do not distinguish between the results of initial and repeat testing, the aggregate data will contain some information bias. The extent and direction of that bias is difficult to predict a priori.

We identified four states in which commercial radon test data and EPA radon data were both available and tested how well the commercial data predicted the EPA data using statistical measures of criterion validity. We report that the commercial radon values were significantly higher than their EPA pairs and significantly overestimated radon at low levels.

## 2. Materials and Methods

### 2.1. Data

Residential radon levels per county were obtained from residential radon surveys conducted by the EPA (Washington DC, USA) using short-term radon tests (charcoal absorption cannisters) placed in the lowest livable area of the residence for 2–7 days [8,12,13,14]. Radon measurements were recorded in pCi/L. Average radon values based on Air Chek test kits (3–7 day test pouches) from the four states (Iowa, North Dakota, Texas, and Wisconsin) at the county level were obtained from the Air Chek (Mills River, NC, USA) website (https://www.radon.com/maps/, accessed on 16 February 2022). Counties with measurements made by the EPA and Air Chek were used in the analyses. Counties with <5 measurements were excluded.

### 2.2. Statistical Analysis

All paired readings from the 4 states were analyzed together, for each state separately, and stratified by high (EPA ≥ 4.0) and low (EPA < 4.0) readings. Values were examined for outliers that could affect the results. Grubb’s outlier test with Rosner’s procedure identified values that were more than 4 standard deviations (SD) from the center of the data.

Paired *t*-tests were used to determine if the average difference of the commercial and EPA readings were significantly different from zero. Significant differences suggest bias (positive or negative) in the commercial readings. Criterion validity, i.e., how well one test corresponds with another, ‘gold standard’ test, was tested for correspondence between the commercial and the EPA data using correlation and regression analyses [15]. Correlation was examined with Kendall’s tau and Lin’s concordance. Kendall’s tau measures the linear association between two numerical variables but, unlike a parametric test, it assumes ranked, ordinal values. Lin’s concordance compares two measurements of the same variable and tests how well a new measurement matches with its pair. Lin’s concordance provides a separate measure of correlation based on the expected value of the square difference (vertical differences between a point and the null line of slope 1 and intercept 0 representing perfect concordance).

Significant correlations suggest a linear pattern of pairwise correspondence between the commercial and the EPA data. Passing–Bablok regression was used to test if the correspondence was biased and linear. Passing–Bablok allows for measurement error in both variables and utilizes the median; thus, distributions do not need to be normally distributed. A linear slope significantly different from one suggests that the correspondence of the two variables changes as the values increase. An intercept significantly different from zero indicates that one measure is consistently higher than the other. NCSS 2020 Statistical Software (2020) (NCSS, LLC., Kaysville, UT, USA, ncss.com/software/ncss, accessed on 11 November 2021) was used for statistical analyses. Alpha of 0.05 was used for all analyses.

## 3. Results

### 3.1. Paired Comparisons

Paired difference values, both commercial and EPA, did not exceed 4 SD from the norm for the combined four states, Iowa, and North Dakota. Texas had only 26 paired values, including one value of 4.16 SD from the norm. That value was excluded. Similarly, Wisconsin had an EPA value of 5.02 SD from the norm which was removed from the Wisconsin subset.

**Figure 1:**
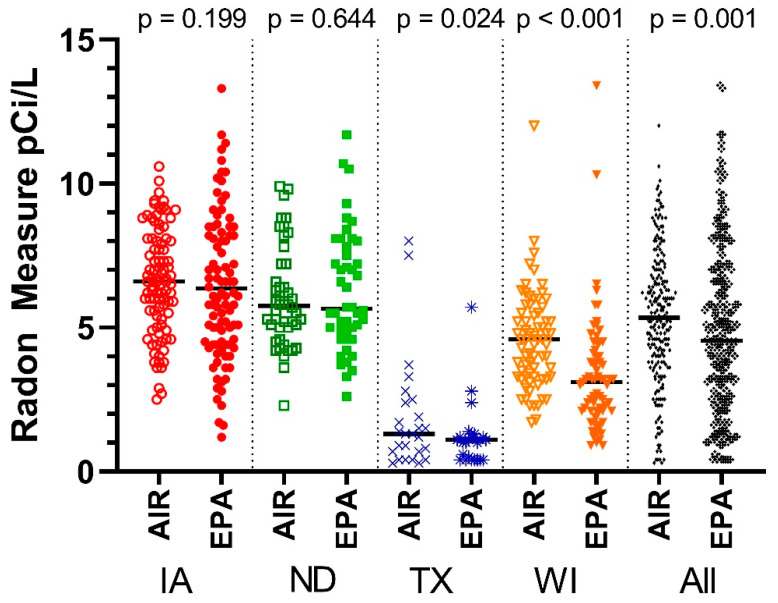
Pairwise comparison of average Air Chek and EPA radon measures by state. Abbreviations: AIR = Air Chek radon measures, EPA = EPA radon measures.

For the 236 readings from counties in all four states, 135 (57.2%) had values ≥4.0. These were separated from counties with lower average values and the analyses were repeated for both groups to determine if the commercial tests performed differently for high and low radon values. For the four states of Iowa, North Dakota, Texas, and Wisconsin, Air Chek radon measures were available for 242 counties, and EPA radon measures for 418 counties. This provided a total of 236 counties with both measures. Figure 1 describes the radon measures by state.

Iowa had 93 paired measures, and the average Air Chek data was not significantly different from the EPA data (*p* = 0.199). North Dakota, with 46 paired measures, also did not differ in average values (*p* = 0.644). Both Texas (*n* = 26) and Wisconsin (*n* = 71) had significantly higher Air Chek measures than EPA (*p* = 0.024, *p* < 0.001). Overall, average Air Chek measures were higher than their EPA pairs.

### 3.2. Criterion Validity

Criterion validity was first tested by examining correlations between the Air Chek and EPA measures from the counties in each state. Table 1 shows the Kendall and Lin’s concordance measures, which test if the paired data had similar patterns. The Air Chek and EPA pairs from the four states were significantly positively correlated (tau = 0.490, Lin = 0.600), although the correlations were not strong (both confidence intervals were under 0.75). A second measure used was the Passing–Bablok regression of Air Chek values predicting their EPA pair. The intercept was estimated at −1.583 (95% CI −2.372 to −0.912), which was significantly lower than zero, indicating that the Air Chek values are biased higher than their EPA pairs. The slope was 1.220 (95% CI = 1.083–1.370), i.e., significantly greater than one. This indicates that although Air Chek values are higher than their EPA values for lower radon values, they are lower than their EPA pairs for higher radon values.

**Figure 2:**
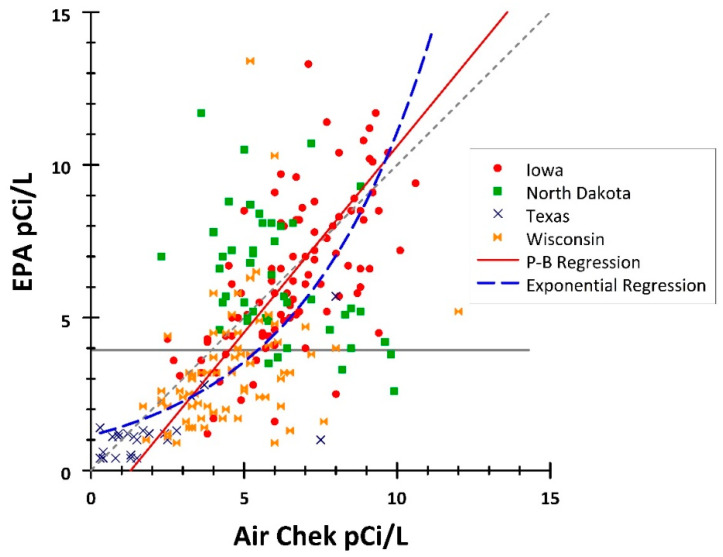
Scatterplot of Air Chek and EPA measures with Passing–Bablok and exponential regressions by state. Abbreviations: P-B Regression = Passing–Bablok regression.

Figure 2 shows the scatter diagram of the Air Chek/EPA pairs for all four states. The null line (grey, dotted line with intercept zero and slope of one) represents a perfect match of Air Chek and EPA values. The Passing–Bablok regression is shown by the solid red line. A CUSUM test of linearity indicated that the values would be fit better by a non-linear estimate (Z = 1.833, *p* = 0.002). The dashed blue line shows the exponential regression $\mathrm{EPA}=1.1405\times e^{\left(0.2273\times \mathrm{Air}\;\mathrm{Chek}\right)}$ fitted to the data.

The criterion validity was then tested for each state (Table 1). All correlations were significantly different from zero. Iowa had the highest correlations (tau = 0.516 and Lin = 0.616). Wisconsin showed the weakest correlations (tau = 0.334 and Lin = 0.278). North Dakota had a negative correlation (tau = −0.241 and Lin = −0.380), suggesting a relationship in which Air Chek and EPA values are reversed (high values match with low values).

The Passing–Bablok regression results for the four states in Table 1 show Iowa, North Dakota, and Wisconsin with negative intercepts, suggesting lower Air Chek readings correspond with higher EPA readings. Texas has a positive intercept, suggesting lower Air Chek values pair with higher EPA values. However, only Iowa and Texas had intercepts significantly different from zero, as evidenced by confidence intervals that did not overlap zero. Iowa and North Dakota had slopes greater than one, though only Iowa’s was significant. That suggests that in Iowa, low Air Chek values correspond with lower EPA values. Texas had a slope significantly less than 1. That suggests that low Air Chek values in Texas correspond with higher EPA values.

As the overall relationship of Air Chek to EPA was not linear, the data were divided between 101 values where the EPA value was <4 and 135 values of ≥4 (see Figure 2). The correlations were slightly less for the measures ≥4 (*n* = 135, tau = 0.259, and Lin = 0.304; Table 1). The intercept for ≥4 was negative (−0.879) but not significantly different from zero, and the slope was positive (1.172) and not significantly different from one. For EPA values <4, the intercept was 0.288 and significantly greater than zero. In addition, the slope was 0.488 and significantly less than one. This indicates that for values <4, the Air Chek values are higher than their EPA pairs.

## 4. Discussion

This study sought to evaluate the validity of commercial radon data in comparison to a “gold standard”, radon measurements reported by the EPA. Criterion validity of the commercial tests at the county level, i.e., how well the commercial data predicted the EPA data for counties within four states, was tested using non-parametric methods. We found that the agreement between the EPA and commercial tests was moderate but was sometimes poor. Specifically, relative to the EPA values, the commercial data were significantly higher for radon values <4.0 pCi/L (the EPA action level for radon remediation). We emphasize that our analyses did not compare the two testers directly: we compared data from the same counties, but not the same houses within those counties. Moreover, the measurements were performed at different times.

What might account for the observed differences in radon values? In addition to the issue of repeated testing of the same house, noted above, possible contributors include intrinsic differences in the accuracy of the test kits, measurement bias, e.g., the commercial tests may systematically measure higher or lower than the EPA test, and temporal trends in radon levels over time. The test results also could differ due to sampling differences in the selection of the homes that were tested.

One factor that could be partly responsible for the lack of agreement is calibration differences between the two sets of radon detectors. However, the Air Chek values were significantly higher than EPA values for low radon levels <4.0 pCi/L (*p* < 0.001) but were lower (though not significantly, *p* = 0.08) for high radon levels ≥4.0 pCi/L. This suggests that the differences are not due to calibration differences *per se*. Conversely, it is possible that the accuracy of at least some commercial tests (although not necessarily the tests sold by Air Chek) may be imperfect. Sun and colleagues compared commercially available, charcoal canister tests from different companies to EPA tests against a known radon standard [16]. They compared 15 detectors from six commercial companies and reported that five companies failed to meet the EPA’s accuracy guidelines (all individual relative errors ≤25%) and four failed to meet precision guidelines (coefficient of variation ≤10% at 4 pCi/L). This suggests that there may be intrinsic differences in the test kits employed by commercial laboratories and the EPA.

Secondly, the data could be influenced by seasonal variations. There is evidence of seasonality in residential radon levels, with higher values in the fall/winter, although not all studies show this [17,18,19]. Although we were unable to find studies on long-term, county-level radon level variability, radon values from CDC’s recent National Environmental Public Health Tracking Network (https://ephtracking.cdc.gov/DataExplorer/, accessed on 14 February 2022) suggest that county-level radon values in the U.S. have not changed markedly over the last few decades. A similar stability of radon levels over time has been reported in Europe. For example, Antignani et al. made repeated measurement of 84 homes in Italy over a 10-year period, using the same methods. They reported that the year-to-year variability of radon measurements made in the same homes was low, with an overall coefficient of variation of 17% [20].

It is likely that some of the differences between the commercial and EPA tests are the result of differences in the selection of homes. Unlike the homes tested by the EPA, the homes tested using commercial test kits are not a random sample. Radon testing rates in the U.S. are known to be higher among better-educated, more affluent homeowners who are more likely to purchase newer, more energy efficient homes. Construction techniques that increase a home’s energy efficiency will result in higher radon levels [21]. For example, Zahnd et al. studied predictors of radon testing for >147,000 radon tests performed in Illinois. Testing rates increased with median home value [22]. A similar positive association between home value and radon testing was reported for homes in Kentucky [21,23]. Thus, it is likely that the higher radon levels reported by commercial labs, at least in part, reflect an over-representation of more expensive homes. As Kendall et al. observed for homes in Great Britain, “ the less affluent have less radon” [24].

Lastly, it is unclear why there was a significant negative correlation between Air Chek and EPA values for North Dakota. Though all North Dakota values fell within the region of other values, they are not in any type of arrangement that leads to a consistent correspondence. The sample size for these paired values is relatively small and the significant inverse correlation thus may be due to chance. Whatever the reason, the commercial values and EPA values accord poorly. Because ecologic studies of radon and disease occurrence generally seek to test a rank order concordance between radon levels and disease rates, this type of bias would bias ecologic studies using commercial radon data toward the null.

## 5. Conclusions

Compared to EPA values for radon tests, we found that the agreement between commercial values and EPA values at the county level is moderate. Commercial tests significantly overestimated radon levels at low values. The use of such data in ecologic studies may introduce confounding due to the over-representation of newer, more expensive homes. We conclude that correlational (ecologic) studies employing radon test results from commercial laboratories should be interpreted with caution.

## Figures and Tables

**Table 1:** Correspondence of Air Chek and EPA radon measures by state for 236 counties.

**Correlation Measures**	**Kendall’s Tau**	**95% CI**		**Lin’s ** **Concordance**	**95% CI**	
		**LL**	**UL**		**LL**	**UL**
Four States (*n* = 236)	0.490	0.423	0.552	0.600	0.516	0.673
Iowa (*n* = 93)	0.516	0.408	0.609	0.616	0.485	0.720
North Dakota (*n* = 46)	−0.241	−0.419	−0.046	−0.380	−0.598	−0.109
Texas (*n* = 26)	0.436	0.188	0.631	0.562	0.336	0.727
Wisconsin (*n* = 71)	0.334	0.187	0.467	0.278	0.094	0.444
≥4.0 (*n* = 135)	0.259	0.150	0.361	0.304	0.148	0.446
<4.0 (*n* = 101)	0.500	0.395	0.592	0.348	0.248	0.440
**Passing–Bablok** **Regression**	**Intercept**	**95% CI**		**Slope**	**95% CI**	
		**LL**	**UL**		**LL**	**UL**
Four States (*n* = 236)	−1.583	−2.372	−0.913	1.220	1.083	1.370
Iowa (*n* = 93)	−3.095	−5.050	−1.232	1.407	1.147	1.688
North Dakota (*n* = 46)	−3.060	−27.90	2.862	1.700	0.567	6.000
Texas (*n* = 26)	0.220	0.121	0.730	0.533	0.200	0.697
Wisconsin (*n* = 71)	−0.993	−2.709	0.404	0.933	0.630	1.364
≥4.0 (*n* = 135)	−0.879	−2.750	0.671	1.172	0.929	1.500
<4.0 (*n* = 101)	0.288	0.169	0.715	0.488	0.394	0.576

Hypotheses being tested: r = 0; CI not including 0 suggests a relationship between Air Chek and EPA. β0 = 0; CI not including 0 suggests Air Chek is biased. β1 = 1; CI not including 1 suggests Air Chek to EPA relationship varies as the values vary. Bolded values indicate measurement name.

## Data Availability

We used publicly available datasets were analyzed in this study. EPA radon data can be found in references [8,12,13,14] and the Air Chek data can be found here: https://www.radon.com/maps/, accessed on 16 February 2022.
